# MicroRNAs as Biomarkers for Early Diagnosis, Prognosis, and Therapeutic Targeting of Ovarian Cancer

**DOI:** 10.1155/2021/3408937

**Published:** 2021-10-21

**Authors:** Yegane Mirahmadi, Reza Nabavi, Fourough Taheri, Mohammad Mahdi Samadian, Zari Naderi Ghale-Noie, Mahsa Farjami, Abbas Samadi-khouzani, Meysam Yousefi, Sara Azhdari, Arash Salmaninejad, Amirhossein Sahebkar

**Affiliations:** ^1^Department of Medical Genetics, Faculty of Medicine, Mashhad University of Medical Sciences, Mashhad, Iran; ^2^Medical Genetics Research Centre, Faculty of Medicine, Mashhad University of Medical Sciences, Mashhad, Iran; ^3^University of Nebraska, Lincoln, NE, USA; ^4^Department of Biology, Faculty of Basic Sciences, Shahrekord Branch, Islamic Azad University, Shahrekord, Iran; ^5^Department of Medical Genetics, Faculty of Medicine, Ahvaz Jundishapur University of Medical Sciences, Ahvaz, Iran; ^6^Department of Anatomy and Embryology, School of Medicine, Bam University of Medical Sciences, Bam, Iran; ^7^Department of Medical Genetics, Faculty of Medicine, Guilan University of Medical Sciences, Guilan, Iran; ^8^Applied Biomedical Research Center, Mashhad University of Medical Sciences, Mashhad, Iran; ^9^Biotechnology Research Center, Pharmaceutical Technology Institute, Mashhad University of Medical Sciences, Mashhad, Iran; ^10^School of Pharmacy, Mashhad University of Medical Sciences, Mashhad, Iran

## Abstract

Ovarian cancer is the major cause of gynecologic cancer-related mortality. Regardless of outstanding advances, which have been made for improving the prognosis, diagnosis, and treatment of ovarian cancer, the majority of the patients will die of the disease. Late-stage diagnosis and the occurrence of recurrent cancer after treatment are the most important causes of the high mortality rate observed in ovarian cancer patients. Unraveling the molecular mechanisms involved in the pathogenesis of ovarian cancer may help find new biomarkers and therapeutic targets for ovarian cancer. MicroRNAs (miRNAs) are small noncoding RNAs that regulate gene expression, mostly at the posttranscriptional stage, through binding to mRNA targets and inducing translational repression or degradation of target via the RNA-induced silencing complex. Over the last two decades, the role of miRNAs in the pathogenesis of various human cancers, including ovarian cancer, has been documented in multiple studies. Consequently, these small RNAs could be considered as reliable markers for prognosis and early diagnosis. Furthermore, given the function of miRNAs in various cellular pathways, including cell survival and differentiation, targeting miRNAs could be an interesting approach for the treatment of human cancers. Here, we review our current understanding of the most updated role of the important dysregulation of miRNAs and their roles in the progression and metastasis of ovarian cancer. Furthermore, we meticulously discuss the significance of miRNAs as prognostic and diagnostic markers. Lastly, we mention the opportunities and the efforts made for targeting ovarian cancer through inhibition and/or stimulation of the miRNAs.

## 1. Introduction

Ovarian cancer is the most frequent type of gynecologic malignancy and it is the fifth leading cause of cancer-related deaths worldwide. According to the estimations, ovarian cancer accounts for 1.3% of all new cancer cases and is the cause of 2.3% of all cancer-related deaths [1]. Despite the administration of standard therapy, which is the combination of debulking surgery and taxane- and platinum-based chemotherapeutic agents, ovarian cancer has witnessed a minimal improvement in the cure rate of the patients. In fact, the average five-year survival of the ovarian cancer patients is less than 50%, highlighting the fact that most of the patients are diagnosed at the advanced stage of the disease [2]. In this regard, studies have indicated that only 15% of the ovarian cancer patients are diagnosed when the tumor is in its early stage and, therefore, most patients are diagnosed after disseminating the tumor to the peritoneal cavity and distant organs. According to the reports, when the tumor is localized to one or both ovaries, ˂10% of the patients succumb to their disease, reflecting the significance of tumor dissemination and metastasis in ovarian cancer mortality [1, 3].

MicroRNAs (miRNAs) are a type of small noncoding RNAs with a length of about 19–25 nucleotides which function in the regulation of gene expression typically through the inhibition of translation and debilitation of messenger RNAs (mRNAs) stability [4]. Therefore, miRNAs control the genes expression correlated with various cellular pathways, including cell cycle regulation, inflammation, cell differentiation, apoptosis, and cell migration. Compelling pieces of evidence have established the role of miRNAs in human cancers. miRNAs dysregulation in human cancers usually occurs through amplification or deletion of miRNA genes, dysregulated epigenetic alterations, defective transcription of miRNAs, and miRNA synthesis machinery [5]. In this regard, the dysregulated miRNAs have been reported as tumor suppressors or oncogenes, augmenting the hallmarks of cancers, including intensified proliferative capacity, resisting cell death signals, and increased cell invasion and angiogenesis [6].

Regarding the high mortality of ovarian cancer which is mostly because of the difficulties in detecting this daunting disease at the primary stage and the paucity of efficacious therapies for patients in the advanced stage or with a recurrent malignancy, the investigation into the role of miRNAs in this cancer pathogenesis and metastasis is highly warranted. Given the fact that an amplitude of mRNAs can be regulated by miRNAs, it is reasonable to expect that a large number of cellular and molecular processes contributing to the formation and dissemination of ovarian cancer are controlled by miRNAs. In this regard, a plethora of studies have explored miRNAs functions in various steps of ovarian cancer, including cancer initiation, progression, and metastasis [7–9]. Understanding the function of microRNAs in the development and metastasis of ovarian cancer will hopefully shed light on finding new diagnostic and prognostic biomarkers for the patients [10–12]. Furthermore, given that miRNAs could act as either tumor suppressor gene or oncogene to regulate gene expression, miRNA-based anticancer therapies are being developed to ameliorate the disease response and cure rate of cancer patients, including ovarian cancer patients, either as a single therapy or with other systemic or targeted treatments [13]. The privilege of miRNA-based therapeutics is in their ability to simultaneously impact various effectors proliferation and tumorigenesis pathways. In this regard, several clinical trials are being recruited to unravel the promising potential of miRNA-based therapies in targeting of ovarian cancer [14].

In this review, we discuss the role of miRNAs in the tumorigenesis and metastasis of ovarian cancer and their use as biomarker for prognostic and diagnostic purposes in this devastating tumor. Furthermore, we illustrate the strategies for the application of miRNA-based therapies in targeting ovarian cancer. We finally present the perspectives, future directions, and the challenges beyond the application of miRNAs in the development of new treatments for targeting ovarian cancer.

## 2. miRNA Biogenesis and Function

miRNAs are small noncoding RNA fragments with the length of 18–24 nucleotides. Their function is to regulate the gene expression. These pieces were first discovered in *Caenorhabditis elegans* in 1990s [15]. Thousands of miRNAs have been detected in a variety of species (i.e., single-cell algae to humans) [15, 16]. More studies have suggested that miRNAs are highly conserved in various species [17, 18]. Bioinformatic evaluations have predicted that miRNAs are expected to control more than fifty percent of the human protein-coding genes [19, 20]. Therefore, given the regulatory effects on different mRNAs, miRNAs play crucial role in numerous cellular processes, including differentiation, cell growth, and apoptosis.

The biogenesis of miRNA begins with the simultaneous or subsequent processing of RNA polymerase II/III transcripts [4, 21]. Around half of the known miRNAs are intragenic, most of which are introns originated and somewhat few are created from protein-coding exons. Left over intergenic miRNAs are transcribed and regulated by specific genes and their promoters [4, 22, 23]. miRNAs are considered as a family when they are transcribed as clusters (i.e., one long transcript) that might have analogous seed areas [4, 24].

**Figure 1:**
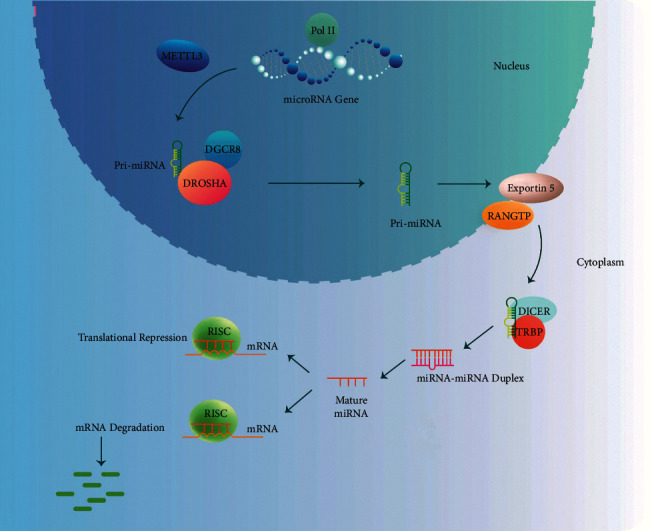
The biogenesis of miRNAs. miRNA genes are usually transcribed by RNA polymerase II, which first produces large transcripts, referred to as pri-miRNAs. The pri-miRNAs are then cleaved by a complex, which is composed of RNA-binding protein DGCR8 and type III RNase, termed Drosha. The resultant ∼85-nucleotide stem-loop structure is pre-miRNA. Following the transportation from nucleus to cytoplasm by Ran/GTP/exportin 5 complex, the pre-miRNAs are cleaved by another RNase III enzyme, which is called Dicer. The miRNA duplexes are then unwound, and the mature miRNA is incorporated into an RISC complex. According to the complementarity between the miRNA and the targeted mRNA, the miRNA-loaded RISC complex mediates gene silencing by cleavage or by a translational repression.

miRNAs biogenesis is carried out through both canonical and noncanonical pathways. The chief pathway involved in the processing of the miRNA is the canonical biogenesis. In this pathway, RNA polymerase II transcribes pri-miRNAs with more than 200 nucleotides in length from related genes, and then, by microprocessor complex, they can be processed into pre-miRNAs. The microprocessor complex is comprised of an RNA-binding protein called DiGeorge Syndrome Critical Region 8 (DGCR8) and Drosha, a ribonuclease III enzyme [4, 25]. Two steps are required for pri-miRNAs to go through the processing. These two processes can be catalyzed by two enzymes of the RNase III family (i.e., Drosha and Dicer). Initially, N6-methyladenylated GGAC and other motifs within the pri-miRNA should be recognized by the complex of Drosha-DGCR8 recognizes [4, 26]; then Drosha slices the pri-miRNA duplex and generates miRNA precursors (pre-miRNA) which are around 70-nucleotide hairpin intermediates. This causes the formation of a 2-nucleotide 3′ that is overhang on pre-miRNA [27]. When pre-miRNAs are created, through a complex called exportin 5 (XPO5)/RanGTP, they can be transferred to the cytoplasm. The next step is the process of them by the RNase III endonuclease Dicer [25, 28] that slashes the transferred pre-miRNAs into ∼22-nucleotide miRNA/miRNA duplexes in cytoplasm. This process encompasses the deletion of the terminal loop and results in a mature miRNA duplex [4, 29]. The name of the mature miRNA form is determined by the direction of the miRNA strand. The 3p and the 5p strands come from the 3′ end and the 5′end of the pre-miRNA hairpin, respectively. Next, the guide strand is integrated into the RNA-induced silencing complex (RISC). Both 3p and 5p strands which are emanated from the mature miRNA duplex can be overloaded to proteins of Argonaute (AGO) family (in humans called AGO1-4) using an ATP-dependent related manner [4, 30]. For each miRNA, the amount of AGO-loaded 3p or 5p strands varies and it highly depends on the type or environment of the cell. This ranges from almost equal proportions to mainly one cell type and environment or the other [4, 31]. A 5 U at nucleotide position 1 or the thermodynamic stability at the 5 ends of the miRNA duplex has a role in selection of 3p or 5p strands in part [4, 32]. Typically, the guide strand is opted according to stability and Uracil content. Therefore, the strand with lower 5 stability or 5 Uracil is preferred to be loaded into AGO. Through the different mechanisms, the passenger strand (i.e., the unloaded strand) will be separated from the guide strand according to the degree of complementarity. This strand of miRNA which has no mismatches is sliced by AGO2. Then it is degraded by cellular machinery that can generate a strong strand bias. miRNA duplexes with central mismatches or non-AGO2-loaded miRNAs are passively disentangled and degraded [4, 21]. miRNAs can aim at the 3 untranslated regions (UTRs) of mRNAs and also can cause degradation of mRNA or translational inhibition of mRNA. Hence, they can suppress protein synthesis and gene expression (Figure 1) [16–18, 20].

So far, several biogenesis pathways of noncanonical miRNA have been explained. In mentioned pathways, various combinations of the proteins engaged in the canonical pathway are utilized. They are predominantly Drosha, Dicer, exportin 5, and AGO2 (Figure 1). Most of the time, the noncanonical miRNA biogenesis is categorized into Drosha/DGCR8-independent as well as Dicer-independent pathways. Of note, pre-miRNAs that are created by the Drosha/DGCR8-independent pathway seem to be the substrates of Dicer. Mirtrons and 7-methylguanosine- (m7G-) capped are examples of such pre-miRNAs. During splicing, they can be generated from the introns of mRNA [4, 33, 34]. Exportin 1 directly contributes to transporting these emerging RNAs to the cytoplasm without cleavage by Drosha. The m7G cap prevents loading of 5p strands into Argonaute that causes a strong bias in the 3p strand [4, 35]. Drosha, instead, processes the Dicer-independent miRNAs from endogenous short hairpin RNA (shRNA) transcripts [4, 36]. In order to complete the maturation of pre-miRNAs within the cytoplasm, they need AGO2 as their length is not long enough for them to be suitable substrates for Dicer. This process consecutively fosters the loading of the complete pre-miRNA into AGO2 and AGO2-dependent slicing of the 3p strand. The maturation process is completed by the 3–5 trimming of the 5p strand [4, 37].

miRNAs have critical roles in numerous types of biological processes such as cell proliferation, differentiation, development, metabolism, migration, and apoptosis through posttranscriptional regulation mechanisms [16–18, 20]. Also, any change in their expression is associated with several human pathologies [20, 38–40]. Moreover, miRNAs can be found in a stable form in plasma and serum samples [20, 41, 42], saliva [43], urine [44], colostrum, tears, peritoneal fluid, seminal fluid, bronchial lavage [4, 45], milk [46], ovarian follicular fluid [47], and cell culture supernatants [48, 49]. Therefore, miRNAs can be found in two stabilized forms in the extracellular spaces. Also, it has been reported that both extracellular and intracellular miRNAs can regulate physiological and pathological events and, therefore, evaluation of their expression profiles can be utilized as potential beneficial markers for the diagnosis of human diseases [20]. Pritchard et al. evaluated circulating miRNAs in 79 solid tumors and noticed that more than half of them were highly expressed in one or more blood cell type. Furthermore, they indicated that miRNA biomarkers in plasma are highly associated with relevant hemolysis (blood cell counts). This observation suggests that the miRNAs in both plasma and serum are mostly stemmed from blood cells [50]. Researchers [51–54] have detected specific kinds of miRNAs enriched in tissues, such as muscle-enriched miR-133, liver-enriched miR-122, and heart-enriched miR-208 in plasma. There are three pathways for their release into the extracellular space: (1) tissue injury, inflammation, and cell death that cause passive leakage from broken cells, (2) microvesicles (MVs) (i.e., active secretion through membrane-enclosed cell fragments) that consist of discarding vesicles and exosomes (under physiological and pathological conditions, almost all cell types that are involved in releasing MVs [20, 55–59]), and (3) active secretion through a protein-dependent MV-free RNA-binding pathway. It is recommended by some researchers [60–63] that numerous RNA-binding proteins that include AGO2, high-density lipoprotein (HDL), and nucleophosmin 1 (NPM1), are capable of merging with miRNAs and bring them outside places of the cells. miRNAs secretion by MVs and HDL-binding is an active process and requires energy in comparison with passive leakage. Active pathways which secrete extracellular miRNAs can mediate regulation of biological processes.

Lately, it is believed that extracellular/miRNAs and those circulating in the blood can be used as biomarkers of various diseases and also play a significant function in cell-cell communications. Not only do these miRNAs have active functions in recipient cells but also a number of them can interact with receptors in cell surface. Therefore, the miRNAs' activities are like hormones. The existence and stability of extracellular miRNAs in circulation indicate an interesting function of these cell-derived miRNAs, but the detailed functions of many secreted miRNAs, especially MV-free, protein-binding extracellular miRNAs, remain to be elucidated. However, in intercellular communication, the extracellular miRNAs seem to be a unique component. Moreover, a new insight can be given by the crosstalk mediated by these miRNAs to unravel the mechanisms of dysfunctionality. Some studies suggest that, during tumor progression, cancer cells dynamically are capable of packaging the miRNAs into MVs and help them to be transferred to the surroundings in order to change tumor microenvironment. Also, it has been found out that extracellular miRNAs can be considered as diagnosis and prognosis biomarkers in various disorders such as cancers [41–44], diabetes, and viral infections [64, 65]. Furthermore, specific genes can be targeted by miRNAs and siRNAs, and these RNAs can regulate the expression of related proteins. Since these RNA molecules can modulate the genes with abnormal expression in different diseases, they may be considered as the promising potential therapy in near future [66, 67].

## 3. The Dysregulation of miRNAs in Cancer

Cancer is a multistep procedure, during which genetic changes in normal cells cause progression of a sequence of premalignant conditions (initiation) into a malignant condition (progression). The resulting cellular phenotype that is transformed has many features. This transformation enables the cells to proliferate independently from growth signals and make them irresponsive to inhibitory signals, elude designed cell death pathways (apoptosis), conquer intrinsic cell replication restrictions, stimulate and maintain angiogenesis, and produce new discontinued colonies with the primary tumor [68, 69]. The leading cause of cancer initiation and progression is dysregulation of genes which are involved in biological processes such as cell proliferation, differentiation, and/or apoptosis. Genes that are correlated with development of various cancers are described as oncogenes and tumor suppressors. Based on function, oncogene products can be classified into six groups: growth factors, growth factor receptors, transcription factors, chromatin remodelers, signal transducers, and apoptosis regulators [69, 70]. Overexpression of the products of these genes provides selective growth advantage that causes tumor growth. The genetic changes can mediate the oncogene activation and result in strengthening the gene, alter promoters/enhancers capability to raise gene expression, or permanently modify protein structure to an active state [70–72]. On the other hand, the products of tumor suppressor genes have regulatory functions in biological processes. Loss or reduced function of tumor suppressors causes dysregulation contributing to cancer [73]. Lately, the description of both tumor suppressors and oncogenes has been changed from the classical protein coding genes and, therefore, various types of noncoding RNAs, like miRNAs, are included in this description [74, 75]. miRNAs play a regulatory role in several cellular and metabolic pathways, especially those that control cell proliferation, differentiation, and survival [40, 76–83]. The dysregulated expression of miRNAs has been indicated in most tumors found [74, 84], but the investigation on grouping of miRNA as tumor suppressors or oncogenes has been quite challenging, since expression patterns of miRNAs have been complicated. For various tissues and differentiation states, the expression patterns of miRNAs differ. Thus, there are two difficulties in the miRNA classification [85–89]. It is sometimes unclear that altered miRNA patterns directly or indirectly contribute to cancer in cellular phenotypes. Moreover, several targets can be regulated by a single miRNA [90]. This phenomenon, as well as tissue specific expression, introduces a single miRNA, either oncogene or tumor suppressor gene, in various contexts.

### 3.1. miRNAs Activity on Cancer and Its Dysregulation

**Figure 2:**
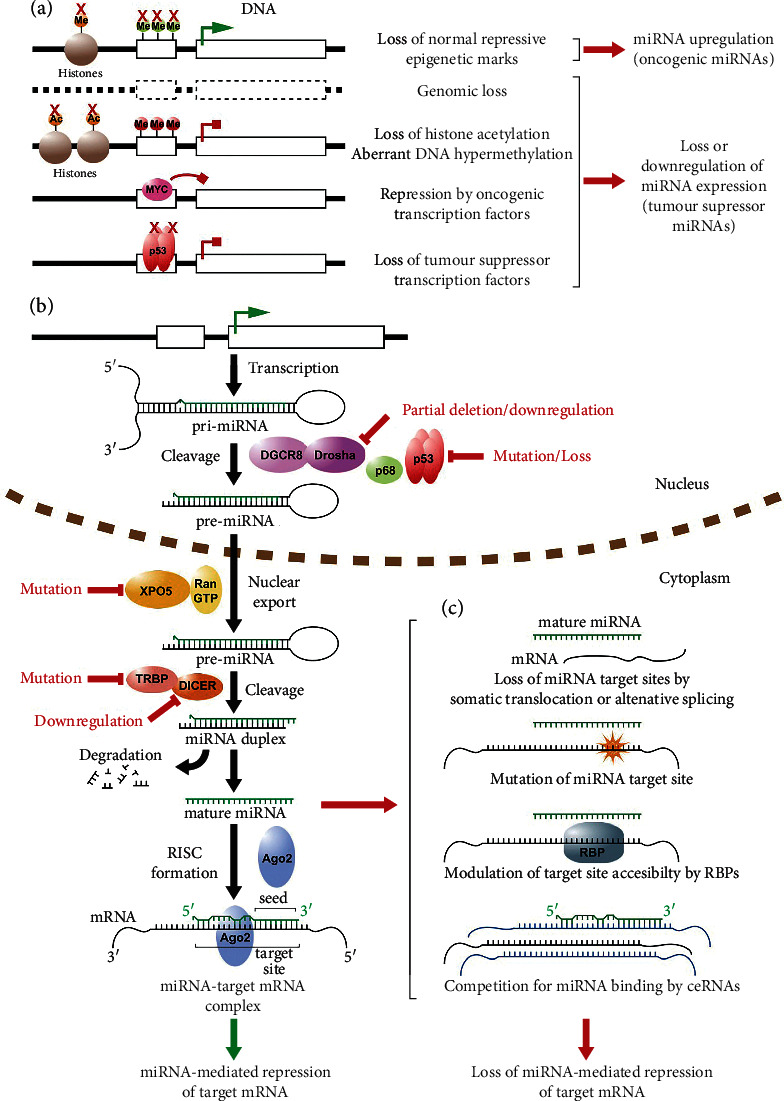
miRNA dysregulation in cancer. A schematic representation depicting the canonical miRNA biogenesis pathway and the general mechanisms whereby normal miRNA expression and function can be dysregulated in cancer. (a) Dysregulation of miRNA gene transcription in cancer through genetic, epigenetic, and transcriptional mechanisms. Active transcription is indicated by green arrow and blocked transcription by red block arrow. Red crosses indicate loss of the gene, epigenetic mechanism, or transcription factor. Me, methylation; Ac, acetylation. (b) Simplified canonical pathway of miRNA biogenesis and processing. Steps commonly dysregulated in cancer are indicated in red. (c) Mechanisms are prevalent in cancer allowing mRNAs to escape regulation by miRNAs. RBP, RNA binding protein. This figure is reprinted with permission from [91] under a Creative Commons Attribution 4.0 International License.

In cancer, the dysregulation of pathway components involved in miRNA biogenesis plus its expression state has been revealed (Figure 2). For example, there is a negative correlation between increase and decrease in the expression of Dicer and Drosha with the advanced stage of tumor and poor clinical outcome. Existing of any flaws in the pathway components of the miRNA biogenesis poses significant change in expression levels of many cellular miRNAs. There are several examples of tumors with global downregulation of miRNA, particularly in differentiated ones. A suggested explanation for this common miRNA downregulation in cancerous cells is that several miRNAs' role is to describe the properties of specific lineages. So, the small abundance of miRNAs raises the undifferentiated state of tumor cells and increases the potential metastasis and invasion of human cancers [88].

According to conducted studies, haploinsufficiency or knockdown of Dicer 1 in rodents has been reported to promote tumor development and progression. This supports the general “tumor suppressor” action of global miRNAs. Conversely, the levels of the processing enzymes such as Drosha and Dicer have increased in tumor cells even though global upregulation of miRNAs is unusual [92–94]. Some examples of dysregulation of various components of the miRNA biogenesis pathway are as below.

### 3.2. Drosha Microprocessor Dysregulation

RNA polymerase II transcribes long pri-miRNAs that endure primary processing in the nucleus by the function of a complex, the Drosha microprocessor, which comprises different cofactors including of DGCR8. In various types of cancers, the upregulation of DGCR8 has been observed. Lately, analyses of gene mutation have indicated frequent heterozygous mutations in the Drosha gene in Wilms tumors [45, 95–99]. It is thought that this mutation interferes with metal binding and destructively regulates the processing function related to Drosha in a main fashion. It reveals a consistency with the global downregulation of miRNAs which is shown in Wilms tumors that harbor mutated Drosha [45, 95–98]. Besides E1147K mutation, numerous other splice-site, missense, and nonsense mutations in Drosha gene have been detected in Wilms tumors, but their effect has not been fully elucidated. Other mutations like somatic as well as germline mutations in DGCR8 have also been observed in Wilms tumors [45, 97, 98]. A “hot-spot” in the first double-strand RNA (dsRNA) binding domain (dsRBDe) of DGCR8 is the missense mutation of E518K, which contributes to over 70% of the DGCR8 mutations in Wilms tumors [45, 97, 98]. The E518K mutation decreases vital miRNAs in tumors [45, 97, 98]. This is in accordance with the fact that DGCR8 knockdown stimulates tumor growth [92]. The expression of alternatively spliced variants of Drosha alongside the gene mutations has been stated in some human cancers, including melanoma and teratocarcinoma cells [100]. The variants of splice site can encode a Drosha protein with a truncated carboxyl- (C-) terminal RNase domain. Its dsRBD is not capable of interacting with DGCR8 and thus is functionally cooperated and functions as a detrimental leader [100]. On the other hand, there is a high copy number of the Drosha gene or overexpression of Drosha protein in over half of the advanced cervical squamous cell carcinomas, which in turn cause a global shift in levels of miRNA [101, 102]. Further studies are needed to reveal why mutations in Drosha gene are often observed in Wilms tumors, besides why Drosha levels are regulated in contrary directions reliant on the tumor type.

Drosha acetylation by CGN5, CBP, and P300 inhibits ubiquitin-mediated degradation and leads to Drosha stabilization [103] and DGCR8 deacetylation by histone deacetylase 1 (HDAC1) increases the resemblance between DGCR8 and pri-miRNAs [104], while phosphorylation of DGCR8 by Erk contributes to stabilizing the DGCR8 protein and promotes miRNA production [105]. It is unclear whether alterations in miRNA modification process resulting from the Drosha PTMs (posttranscriptional modifications) are practical in cancer. In contrast, both HDAC1 and GSK3B are regularly dysregulated in cancer; thus it is probable that various Drosha PTMs may be observed in cancer cells in comparison with nontumor cells.

Several nuclear proteins can play a role in regulating the activity of Drosha in a way that it commonly influences the biogenesis of only a small subset of miRNAs. An example of such nuclear protein is adenosine deaminase acting on RNA (ADAR). ADAR is an RNA-editing enzyme that transforms adenosine (A) to inosine (I) in double-stranded RNAs. ADAR1 constructs a complex with DGCR8 and inhibits the activity of Drosha [106]. The level of ADAR1 decreased in metastatic melanoma, and two miRNAs (miR-17 and miR-432) are excessively produced and stimulate tumor development [106]. Also, Smad proteins, which are the signal transducers of the transforming growth factor-*β* (TGF-*β*) family of growth factors, mediate the change in Drosha activity in the nucleus. While Smad proteins were predominately cytoplasmic at even state, they are translocated to the nucleus during ligand activation.

Smad proteins are inherent transcription factors with DNA attachment and transcription activating domains [107]. Nevertheless, these proteins are enlisted for the complex of Drosha microprocessor via physical interaction with the RNA helicase p68, also identified as DDX5. Pri-miRNA is escalated here to pre-miRNA processing of approximately 20 miRNAs [108], which include miR-21 and miR-199. The studies have shown that miR-21 as an oncogenic miRNA (onco-miR) is frequently upregulated in roughly all tumor samples and is recognized as an inhibitor of a large number of tumor suppressor genes such as *PDCD4*, *PTEN*, *SPRY1/2*, *TPM1*, and *TP53BP2* [109]. Furthermore, miR-21 is able to lead tumorigenesis by preventing the negative regulators like Ras/MEK/Erk pathway [110].

The regulation of pri-miRNA processing mediated by R-Smad is obtained by direct connection of R-Smads with a sequence component in the stem district of pri-miR-21 [111]. In addition, similar to Smads, p53 can provoke the expression of several suppressor miRNAs (suppressor-miRs) (miR-143, miR-15/16, miR-203, and miR-145) by linking with the complex of Drosha microprocessor through p68 and expediting pri-miRNA processing [112]. Although genotoxic stimuli induce acetylation of K120 in the DNA binding domain of p53, they do not influence the transcription activity of p53. They lead to the correlation of p53 with the complex of Drosha microprocessor and promote miR-203 quantities to increase apoptosis as a replacement for cell cycle arrest [113]. It is not clear yet how p53 can regulate the processing of a specific subset of miRNA and how activation of Smads mediated by TGF-*β* can influence the process of miRNA regulation mediated by p53.

A new research also detected Yes-associated protein (YAP), a signal transducer of the Hippo pathway, which monitors the size of organ by sensing cell density, as a regulating factor of Drosha microprocessor activity [114]. In normal or cancerous cells at low cell density in which Hippo signaling is not active, YAP is collected in the nucleus and is capable of binding to the RNA helicase p72 (also known as DDX17) and then sequestering it from the Drosha microprocessor, which can inhibit a subset of miRNAs that aim at Myc [114]. There is a possibility that the inhibition of Drosha by YAP/p72 complex may play an important role in the common downregulation of a large group of miRNAs in cancer. The single-strand RNA binding factor KH-type splicing regulatory protein (KSRP, also known as FUBP2) combines to a small subset of pri-miRNAs through their terminal loops. This includes onco-miRs in the let-7 family, miR-21 and miR-125, and stimulates processing by Drosha by an unidentified mechanism [115, 116]. When the PI3K/Akt signaling pathway is activated, KSRP is phosphorylated at the sites of S274 and S670, which triggers enhanced binding to pri-miRNAs and induces their Drosha-dependent processing [115]. Moreover, DNA damage leads to activation of KSRP-enhanced miRNA biogenesis [117]. In comparison with the chronic phase disease, KSRP is exceedingly expressed in chronic myeloid leukemia (CML) acute phase/blast crisis. However, it is not clear whether changed expression or function of KSRP aids in leukemogenesis mediated by dysregulation of miRNA processing [118].

Some other RNA binding proteins, like hnRNP A1, serine/arginine-rich splicing factor 1 (SRSF1), and FUS (also known as TLS), have been discovered to be related to pri-miRNAs and assist Drosha processing, but the direct contribution of them in tumorigenesis has not been evidenced. Modification of pre-miRNA export, dysregulation of the Dicer/TRBP processing complex, and posttranslational modifications of Ago proteins can be other reasons for dysregulation, which are described further in [95].

## 4. miRNAs and the Pathogenesis and Metastasis of Ovarian Cancer

Ovarian cancer cells could originate from epithelial, stromal, or germline cells, among which epithelial tumors are the most common type [119]. Demographic and lifestyle factors, ethnicity, advanced age, and female reproductive hormones are the risk factors associated with ovarian cancer. However, a positive family history of ovarian, uterine, breast, or colon cancer is linked to mutations in *BRCA1*, *BRCA2*, or *TP53* genes, the most potent risk factor [120]. At the molecular level, ovarian cancer is considered to be an extremely heterogeneous disease with somatic and germline mutations, in tumor suppressor genes and DNA repair genes, and oncogenes such as *BRCA1*, *BRCA2*, *KRAS*, *BARD1*, *BRAF*, *PTEN*, *PIK3CA*, *BRIP1*, *MRE11A*, *MSH6*, *CHEK2*, *NBN*, *RAD50, PALB2*, *RAD51C*, and *TP53* have role in proliferation, invasion, and metastasis process of ovarian cancer cells [121]. Alongside genetic mutations, alteration in the expression of these genes and other genes related to carcinogenesis may result in the aberrant proliferation of ovarian cancer cells.

As mentioned earlier, miRNAs are determined to regulate up to half of activities of all protein coding genes [122] and are observed to manage almost all the cellular processes [123]. Several studies discovered that miRNAs' dysregulation leads to different human diseases like cancer [124, 125]. Due to their involvement in oncogenesis, miRNAs function as either tumor suppressors or oncogenes, according to their function and expression pattern [126]. High-throughput technologies indicated that miRNAs have a fundamental effect on cancer-specific targets in all cancers [38, 127]. Broad dysregulation of miRNAs ultimately causes cancer. There are altered mechanisms that are responsible for this circumstance: DNA point mutations, epigenetic mechanisms, translational modulations, chromosomal alterations, and genetic and epigenetic modifications in various stages of transcriptional and posttranscriptional pathways [128]. Low activities of Dicer and Drosha are closely correlated with end-stage ovarian cancer and low suboptimal surgery, respectively [124]. The upregulation and dysregulation of miRNA in ovarian cancer are key features of malignancies regarding tumor suppressor genes or oncogenes [40]. The miRNAs presence in serum is associated with the occurrence of malignancies and tumors and has also been used as a diagnostic tool for detecting cancers [129].

Numerous studies confirmed that approximately 40% of the miRNA genes reveal modified DNA copy numbers [130, 131]. The worldwide miRNA expression of human ovarian cancer and the differential expression in cancer cells and normal ovarian tissue have been investigated by Iorio et al. which revealed the upregulation of miR-200a, miR-141, miR-200b, and miR-200c in carcinomas and the downregulation of miR-125b1, miR-145, miR-140, and miR-199a. The deletion of miR-140 in ovarian cancers leads to the belief that it is related to invasion, involving matrix metalloproteinase-13 (MMP-13), fibroblast growth factor 2, and angiogenic VEGF-A [132]. miR-138 downregulates the expression of SOX4 and HIF-1a and, consequently, suppresses ovarian cancer invasion and metastasis. These are more involved in the regulation of EGFR and Slug pathways, respectively [133]. The overexpression of miR-320 was reported to be tangled in invasion and metastasis in ovarian cancer [134]. Similarly, the overexpression of miR-196a stimulates epithelial ovarian cancer (EOC) to migrate [135].

In ovarian cancer, miRNAs' involvement in various cellular pathways includes cell cycle, tumorigenesis, proliferation, apoptosis, invasion, and metastasis. The expression profiling of miRNAs in various cancer types revealed their pivotal roles in recognizing the miRNAs' function in ovarian cancer. It has been shown that miRNAs are oncogenes and tumor-suppressor genes; hence, they can be considered reliable biomarkers for ovarian cancer. miRNAs are more specific and sensitive than any other biomarkers and their effectiveness is worth to be scrutinized for the diagnosis and prognosis of ovarian cancer [39, 136]. Some significant miRNAs that have a role in the pathogenesis and metastasis of ovarian cancer are listed in Table 1. The following is a concise overview of some important miRNAs associated with ovarian cancer's pathogenesis and metastasis processes.

### 4.1. miR-182

miR-182-5p is an oncogenic miRNA positioned on 7q32.2 region and its overexpression is prevalent in many solitary malignancies, including melanoma [155], colon cancer [156], breast cancer [157], and ovarian cancer [155, 158]. miR-182 is one of a few miRNAs affected by cellular stress or damage to DNA [19], and, therefore, its overexpression results in tumor development. The oncogenic features of miR-182 in ovarian cancer are noticeably due to impairment in double-strand breaks repair in DNA, negative control of BRCA1, suppression of metastasis suppressor 1 (MTSS1), and overexpression of high-mobility group AT-hook 2 (HMGA2) [155, 159–162]. MTSS1 and HMGA2 may represent a central role in miR-182-mediated tumor metastasis. HMGA2 protein, encoded by the HMGA2 gene, has different effects on a number of biological processes, including responses to DNA damage repair, cell cycle, apoptosis, cell senescence, epithelial-mesenchymal transition (EMT), and telomere restoration, so that its aberrant expression in adult tissues is generally associated with both malignant and benign tumor formation [163]. Recent investigations showed that HMGA2 is significantly overexpressed in most of ovarian cancers [164]. Multiple studies have reported that miR-182 enhanced HMGA2 expression and promoted ovarian cancer invasion via direct binding to the SNAIL1 promoter. SNAIL1 is a zinc-finger transcription factor playing critical roles in tumor progression and EMT [165, 166].

Metastasis suppressor 1 (MTSS1), which is also referred to as missing-in-metastasis (MIM), is a potential metastasis suppressor protein [165], which functions as a cytoskeletal scaffold protein and influences cytoskeletal movements through interacting with Rac, actin, and actin-associated proteins [167]. Stress fiber formation (F-actin) is one of the most critical events of cytoskeleton rearrangement during migration and invasion of cancer cells blocked by MTSS1 (27). MTSS1 is considered as a top target of miR-182 and, therefore, is significantly downregulated in many metastatic types of cancer [162].

Discoidin domain-containing receptor 2 (DDR2) is another miR-182 target gene that mediates cell interactions of extracellular matrix (ECM) so may contribute to cancer cell proliferation and migration [168–170]. DDR2 affects the SNAIL1 function in ovarian cancer and contributes to invasion and cell migration [171]. Ramalho et al. have revealed that the overexpression of DDR2 leads to decreased miR-182 levels and worse progression-free survival. Accordingly, these molecules might be related to cancer progression [172].

DCN (Decorin), AKT3 (AKT Serine/Threonine Kinase 3), and TIMP2 (tissue inhibitor of metalloproteinases 2) are the other novel candidate target genes for miR-182 involved in the regulation of ovarian cancer biological processes [173]. Due to the above statements, blocking the expression of miR-182 will rescue some well-known genes suppressing the tumor and recover the cell's critical biologic functions.

### 4.2. miR-200 Family

There are five miRNAs (miR-141, miR-200a, b, c, and miR-429) in miR-200 family and they are abundantly expressed in epithelial tissues. miR-200b/a and miR-429 are on chromosome 1p36.33 and miR-200a/141 are located on different clusters of chromosome 12. miR-200c can promote CD44 expression. CD44 is a multifunctional molecule which is involved in cell-to-cell interactions, adhesion, and migration. Consequently, decreased expression of miR-200c could lead to metastatic ovarian cancer [174]. Due to its dual function in various cancer cells, miR-429 is a tumor suppress in bladder cancer [175], gastric cancer [176], and cervical cancer [177] and has an oncogenic role in colorectal and ovarian cancers. Plenty of studies indicate that miR-429 has a fundamental role in the initiation and promotion of EOC through the regulation of EMT. In this regard, it has been identified that promoter hypermethylation of miR-429 suppressed its expression and therefore upregulated its corresponding target genes, including *KIAA0101*, *ZEB1*, and *ZEB2* [178].

**Figure 3:**
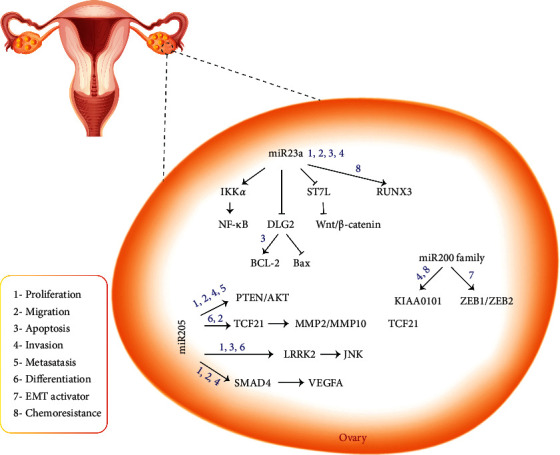
This schematic figure represents the main miRNAs and gene targets involved in the biological processes of ovarian cancer. miR-200 family are abundantly expressed in epithelial tissues. This miRNA induces the expression of CD44, which plays a key role in cell-to-cell interactions, adhesion, and migration. The cluster of the miR-23a is involved in the initiation and progression of ovarian cancer through affecting transcription factors like NF-*κ*B and WNT pathway, regulating the transcription of various genes involved in cytokine production, cell survival, proliferation, and differentiation. miR-205 targets many biological processes like proliferation, differentiation, and migration and facilitates apoptosis in some ovarian cancer cells.

It has been identified that ZEB1, as a mesenchymal marker repressing the transcription of E-cadherin, is an EMT activator [179]. Indeed, inhibiting ZEB1 and ZEB2 upregulates E-cadherin and reduces cell motility [180]. ZEB1 and ZEB2, as targets of miR-429, inhibit this regulatory miRNA and alter the expression of the calcium-dependent adhesion protein E-cadherin [181, 182], inducing EMT in various cell types [183] (Figure 3).

KIAA0101 protein (PCNA-associate factor) is a cell cycle-regulated protein occurring most abundantly during S/G2 phase and acts as a regulator of DNA repair during DNA replication (42). KIAA0101 is required for the EOC cell invasion and chemoresistance (Figure 3) and is the target gene for miR-429. Therefore, decreased expression of miR-429 in the metastatic cells in EOC tissues leads to the upregulation of KIAA0101 and enhances the migratory activity and chemoresistance of EOC cells [184].

### 4.3. miR-23a

miR-23a belongs to the miR-23∼27∼24 cluster in 19p13, where the miR-23a-27a-24-2 cluster is located [185]. The transcription of this cluster results in the production of a pri-miRNA transcript consisting of miR-23a, miR-27a, and miR-24. miR-23a, as an oncogenic miRNA (onco-miR), has multiple-gene regulatory functions and is significantly increased in different cancer tissues such as bladder cancer, glioblastoma, pancreatic cancer, and gastric cancer [186–188]. Moreover, this miRNA is involved in the initiation and progression of a variety of cancers [189].

Ikappa B kinase-alpha (IKK*α*) and suppression of tumorigenicity 7 like (ST7L) are two miR-23a target genes that are shown to provoke the oncogenic role of miR-23a and contribution in the malignancy of EOC cells through effects on nuclear factor kappa B (NF-*κ*B) and Wnt pathways [190]. NF-*κ*B transcription factor acts as a protein complex regulating the transcription of various genes involved in cytokine production, cell survival, proliferation, and differentiation [191]. IKK*α* is one of the NF-*κ*B pathway proteins that activated NF-*κ*B to modulate target gene expression [191]. ST7L is a suppressor of the Wnt/*β*-catenin signaling pathway and acts as a tumor suppressor in many cancers [192]. For the control of cell proliferation, migration, and apoptosis as well as the promotion of EMT by inducing the expression of EMT transcription factors, the Wnt/*β*-catenin signaling pathway is essential [193, 194]. Yang et al. reported that, by binding to their 3′-untranslated regions (3′-UTRs) in EOC cells, miR-23a might increase IKK*α* expression but decrease ST7L expression. Regarding IKK*α* as an essential factor for the NF-*κ*B pathway and ST7L as a Wnt pathway inhibitor, miR-23a positively regulates these pathways inducing cell growth, migration, and invasion in EOC cells [190] (Figure 3).

Discs large MAGUK scaffold protein 2 (DLG2) is found to be another miR-23a target. DLG2 is a member of the membrane-associated guanylate kinase (MAGUK) family, which has various protein-protein interaction domains having a role in a broad range of cellular pathways [195]. miR-23a can inhibit its expression through binding specifically to the DLG2 gene and inhibit its expression. Zhuang and his colleagues found that miR-23a, via targeting the DLG2 expression, elevates antiapoptotic Bcl-2 expression and suppresses proapoptotic protein Bax expression, blocking ovarian cancer cell apoptosis and inducing tumor stem cells development (Figure 3) [196].

miR-23a is also related to the chemotherapy-resistant ovarian cancer cell lines [197]. The *RUNX3* gene is one of the antioncogenes that have a quenching effect on multidrug resistance gene 1 (MDR1) expression. MDR1 acts as a drug pump to excrete the chemotherapeutic agents out of the cells (Figure 3). One of miR-23a targets is the 3′-UTR of *RUNX3* gene [198]. Jin and Wei by studying on drug-resistance ovarian cancer A2780 cell lines indicated that, in the presence of a highly expressed level of miR-23a, the silenced gene MDR1 was expressed, and the classic resistance mechanism was initiated [199].

### 4.4. miR-205

Human miR-205 is a highly conserved miRNA placed on chromosome 1q32.2. miR-205 has dual roles in cell regulation due to its involvement in both physiological and pathological pathways by targeting oncogene or tumor suppressor genes [200]. According to the various evidences, miR-205 is elevated in a broad range of cancers such as lung [201], kidney and bladder cancers or prostate [202], esophageal [203], melanoma [204], and breast [205] cancers. In EOC cells, *SMAD4* and *PTEN* (phosphatase and tensin homolog) genes have been identified as miR-205 targets, and their downregulation contributes to cell proliferation, migration, and cancer cell invasion [206]. Smad4 (SMAD family member 4) is activated through phosphorylation by transmembrane serine-threonine receptor kinases in response to TGF-*β* signaling and regulates the transcription of target genes related to cellular adhesion, motility, and differentiation. Therefore, the Smad4 reduction correlates the cancer progression [207, 208]. *PTEN* tumor suppressor gene is located on 10q23.3 and is altered in various cancers at high frequency. Various studies have confirmed its ability in decreasing tumor growth and chemoresistance via blocking various cell signaling pathways [209]. Loss of *PTEN* expression in several tumor types indicated that low PTEN expression might be an accelerating factor in the development of cancer cells. Shi and his coworkers found that the downregulation of PTEN/AKT pathway via miR-205 is contributed to cisplatin-resistant C13K ovarian cancer cells [210]. Furthermore, findings from the research of He et al. show that exosomal miR-205 significantly accelerated angiogenesis and promoted metastasis in a mouse model and HUVECs human cell lines by targeting PTEN-AKT pathway [211] (Figure 3).

Leucine-rich repeat kinase 2 (LRRK2), as a target of miR-205, has been introduced in another study of Liu et al. They showed that LRRK2 has lower expression in ovarian cancer tissues than in healthy ovarian tissues [212]. LRRK2 is a kinase mediating the c-Jun N-terminal kinase (JNK) phosphorylation [213]. The JNK signaling pathway participates in biological processes of proliferation, differentiation, and apoptosis and facilitates apoptosis in some ovarian cancer cells [214]. Therefore, decreased LRRK2 expression by miR-205 results in the JNK pathway inactivation and the apoptosis inhibition of ovarian cancer cell. VEGFA (vascular endothelial growth factor A) is known as another target gene of miR-205 increasing the ovarian cancer risk by targeting EMT progression factors [215]. Another direct target of miR-205 is transcription factor 21 (TCF21), which is a reduced tumor suppressor in ovarian cancers [216]. TCF21 is an essential factor in epithelial cell differentiation which is located on chromosome 6q23 [217]. MMPs are involved in tissue cell integrity through the regulation of cell-matrix composition and play a pivotal role in tumor progression [218]. TCF21, as a transcription factor, is reported to decrease MMP-2 and MMP-10 expression and lead to the maintenance of cell integrity. In ovarian cancer, miR-205 directly targets TCF21, resulting in high expression of MMP-2, which increases cancer cell migration [216, 219] (Figure 3).

## 5. miRNAs as a Biomarker for Prognosis and Early Diagnosis of Ovarian Cancer

One of the most common gynecologic malignancies in women is ovarian cancer and the patients show symptoms even at the primary stages of cancer. These symptoms are regularly misunderstood as they are closely associated with other gastro- and gynecological diseases leading to the misdiagnosis of this disease [220, 221]. Around 230,000 new plus 140,000 death cases are reported annually [202]. Generally, ovarian cancer starts from granular-theca and germ cells. Also, more than 90 percent of patients have epithelial histology stemming from surface covering cells or subsurface cyst cells [222]. EOC is the most common cancer type compared to the other stromal and germline tumor types [119, 221]. A family history of cancer, age, and repeated ovulation with mutation in BRCA1 are associated with ovarian cancer [223]. Even though there are advancements in the identification of cases and cytotoxic treatments, only one-third of patients with an advanced stage of cancer live approximately five years after prognosis [224], whereas the mortality rate of the rest of the patients is high because of diagnosis at the late stage [132]. Moreover, about 19% of ovarian cancer patients are diagnosed at the early stages of cancer [132]. Genetic and epigenetic alterations both help in the progression of ovarian cancer which can be created from gene amplification, deletion, and changes in the methylation state [221, 225]. Ovarian cancer is of two types of tumors: low-grade and high-grade types that are based on gene mutations. Low-grade group is less common. These are diagnosed at early stages, and tumor growth is stepwise and encompasses mutations in KRAS, BRAF, PIK3CA, and PTEN. High-grade tumors are more common, which are mostly diagnosed at late stage. They are aggressive in nature and include mutations in TP53, BRCA1, and BRCA2 [221, 226, 227]. TP53 mutations are the most typical in ovarian cancer, whereas inactivation of phosphatase and tensin homolog (PTEN) is less typical. Mutations in DNA repair genes *BRCA1* and *BRCA2* contribute to heredity to ovarian cancer and accordingly increase the genomic instability [228]. At molecular levels, ovarian cancer has a highly heterogeneous disease etiology [229]. As a consequence of inadequacy of standard diagnostic and pelvic tests, serum cancer antigen 125 (CA125), and transvaginal ultrasonography for the detection of cancer at the early stage, there are more cases of death [124]. The main root of poor prognosis can be due to subtle disease symptoms at early stages, chemotherapy resistance, and lack of precise noninvasive detection methods [132, 220]. Surgery and chemotherapy are preferred treatments for advanced ovarian cancer, and, even with chemotherapy advancements, there is the possibility of chemotherapy resistance or relapses. Ovarian cancer diagnostic methods consist of pelvic examination, transvaginal ultrasound, and CA125 measurements [130]. So, advanced methods for detecting ovarian cancer, especially at early stages, are critical for prescription of the right medications and therapies. Clear cell, endometrioid, serous, and mucinous, with serous as the most frequent type, are the four major histological subtypes of ovarian carcinoma [132]. Analysis of the newest large-scale data of ovarian cancer samples recommends invasiveness at mesenchymal subtype of tumors to be connected with TGF-*β*. TGF-*β*, a multifunctional protein, stimulates EMT, resulting in metastasis and chemotherapy resistance in various kinds of cancers [230]. These histological types are related to different morphologic and genetic modifications [231]. Recently, expression profiling technologies have been significantly advanced, which expand the awareness on molecular implications of cancer in order to utilize them for diagnosis, therapy, and cancer drugs development. Therefore, the innovative and superior markers and drugs for diagnosis and treatment of ovarian cancer are figured out. Several researchers have recognized and examined modified expression of miRNAs in ovarian cancer, which results in the establishment of macrometastasis. miRNAs are used as novel diagnostic and prognostic markers and could be considered as novel targets for therapeutic purposes [232] (Table 2).

### 5.1. The Patterns of miRNA Expression in Ovarian Cancer

Numerous experiments of miRNA and cDNA microarrays in ovarian cancer have shown large transcriptional variations [233]. Various miRNAs are indistinguishable to be downregulated in miRNA expression profiles of ovarian cancer, validating miRNAs' role in tumorigenesis [124]. Iorio et al. compared the expression profiles of miRNAs between ovarian cancer tissues or cell lines with normal tissues and showed that only 29 miRNAs, miR-14, miR-200a, miR-200b, and miR-200c were found to be upregulated in cancer cells, and there is a downregulation in the rest of all the 25 miRNAs comprising miR-140, miR-145, miR-199a, and miR-125b-1 [132]. They also mentioned that there is a distinction between the miRNA signatures in different histotypes of ovarian cancer. In another study by Vang et al., miRNA expression patterns of primary serous ovarian cancer and their omental metastasis had significant differences, measured by qPCR. miR-146a and miR-150 were related to omental metastases, cisplatin resistance, and spheroid formation [234]. Also, Calura et al. showed the profile of miRNA expression of each stage I EOC histotype and revealed the overexpression of miR-30a-5p and miR-30a-3p on clear cell type and the higher level of miR-192 and miR-194 on mucinous histotype [235]. Furthermore, Nam et al. showed the upregulation of 11 miRNAs (miR-16, miR-20a, miR-21, miR-23a, miR-23b, miR-27a, miR-93, miR-141, miR-200a, miR-200b, and miR-200c) and downregulation of 12 (miR-10b, miR-226a, miR-29a, miR-99a, miR-100, miR-125a, miR-125b, miR-143, miR-145, miR-199a, miR-214, and let-7b) through the miRNA microarray of 20 serous ovarian carcinomas and their comparison with normal samples [236].

The Cancer Genome Atlas (TCGA) project has revealed the mRNA and miRNA expression, promoter methylation, and DNA copy number in 489 patients with high-grade serous ovarian adenocarcinomas [237] and it has been stated that TP53 mutation has been detected in nearly 96% of tumors plus recurring somatic mutations of NF1, RB1, CDK12, BRCA1, and BRCA2 [124]. Eight key miRNAs (miR-141, miR-182, miR-200a, miR-506, miR-25, miR-29c, miR-101, and miR-128) were found and thought to target up to 90% of its network [124]. A combination of genomic methods, including array-based comparative genomic hybridization (aCGH), miR microarray, cDNA microarray, and tissue array, was utilized to evaluate alterations in the miRs' expression in EOC [233]. It was discovered that genomic losses along with epigenetic variations might cause the downregulation of miRNA. From 35 dysregulated miRs in ovarian cancer (immortalized ovarian surface epithelial cells), 31 (88.6%) had almost lost their expression in comparison with normal cells, including the miRs that are tumor suppressors, like let-7d [238] and miR-127 [239].

Experiments also disclosed that miR-199a, miR-214, miR-200, miR-100, and let-7 family are the most extremely expressed candidates in normal ovarian cells and EOC in different ways [130, 202, 240]. The expression of miR-200 family detected in epithelial tissues includes five members (miR-200a, miR-200b, miR-200c, miR-141, and miR-429). These miRNAs are divided into two groups in the human genome. miR-200a, miR-200b, and miR-429 are grouped on chromosome 1, while miR-200c and miR-141 are found on chromosome 12 [241]. Numerous researches have indicated variations in the expressions of distinct components of the miR-200 family and exhibited their essential functions in ovarian carcinoma development. These experiments showed the miR-200 family in three categories of ovarian cancer: serous, endometrioid, and clear cell carcinomas. However, they revealed the upregulation of miR-200b and miR-141 merely in endometrioid and serous categories. This recommends a more intricate and distinct role of the miR-200 family in ovarian cancer than it was believed primarily [132]. Therefore, different miRNAs have been identified as vital prognostic and diagnostic biomarkers that can be served as a potential utility in ovarian cancer.

### 5.2. miRNAs Alterations in Ovarian Cancer

The copy number variations of genes that are near cancer-associated genomic regions (CAGRs) influence miRNA expression [259]. A comprehensive study of cancer modifications was presented with aCGH to recognize miRNA loci that are gained or lost in some human cancers, including ovarian cancer, breast cancer, and melanoma [131]. Zhang et al. studied 283 miRNA loci and discovered that 37.1% of these miRNAs had significant alterations in their copy number [131]. Similarly, substantial copy variations were noticed in 72.8% of breast cancer samples and 85.9% of melanomas samples. The miR-15a/16-1 locus is lost in up to 24% of ovarian cancer samples and miR-17-92 in all tumor tissues [123]. A copy number increase in Dicer1 and Ago2 loci has also been reported in, respectively, 24.8% and 51.5% of ovarian tumor regions [131]. As stated earlier, an effectual miRNA processing and operation requires Dicer and Ago2 proteins. Short-hairpin-RNA- (shRNA-) mediated knockout of Dicer1 and Ago2 boosts colony development in both *in vivo* and *in vitro* environments [92]. Modifications in Dicer1/Ago2 expression result in huge alterations of miRNA expression, which commonly occur in cancer through either germline deletions, mutations, or promoter methylation [239, 260]. Mutation of p53 is also one of the most widespread changes noticed in EOC, especially in high-grade serous tumors [261].

### 5.3. Dysregulation of miRNA in Ovarian Cancer

Many experiments on the expression of miRNAs in ovarian cancer and normal epithelial ovarian cells as well as immortalized ovarian surface epithelial cells [262–264] have reported the dysregulation of 310 miRNAs in ovarian cancers, among which 34 miRNAs were constantly dysregulated in carcinoma tissues in at least three separate studies. Numerous miRNAs discovered to control growth of ovarian cancers are miR-31, miR-34abc, miR-125b, miR-127, and let-7a/b/d/f miR-31 [265]. According to studies, oncogenic miR-182 expression is upregulated in high-grade ovarian tumors, resulting in proliferation and progression of cancer cells by enhancing the BRCA1/HMGA2 dysregulation [266]. miR-221-3p plays a tumor suppressor role by affecting ARF4 to repress the proliferation and migration of EOC cells. Those having the high miR-221-3p expression can survive better as compared to others [267]. miRNAs are the regulators of chemosensitivity, since dysregulated miRNA expressions contribute to therapeutic chemoresistance. The upregulation of miR-106a and downregulation of miR-591 cause chemoresistance to Paclitaxel, so the chemosensitivity of Paclitaxel can be achieved by downregulation of miR-106a and upregulation of miR-591 [268]. miR-145 also sensitizes the resistant ovarian cell to Paclitaxel by regulating the Sp1 and Cdk6 [185, 269].

### 5.4. miRNAs as Prognostic Markers in Ovarian Cancer

For ovarian cancer diagnosis and detection, the miRNAs can be utilized as an essential tool. Downregulation of miR-9 has been shown in ovarian cancer cases [270]. Upregulation of miR-92, miR-21, and miR-15a has been proposed to be a signature of ovarian cancer. The underexpression of miR-31 indicates the initial phase of ovarian cancer growth [265]. On the other hand, the downregulation of miR-34 a/b/c/miR-449b, miR-503, and miR-507 has been noticed in patients of last phases [233, 271, 272]. Research has also shown the overexpression of miR-200 family and underexpression of let-7 family in EOC cases. In this regard, it has been reported that serum miRNAs, miR-21, miR-155, and miR-210, were significantly higher in cancer patients compared with normal participants [273]. Chung et al. showed let-7b, miR-26a, miR-132, and miR-145 as diagnostic markers in EOC patients [274]. Zheng et al. also indicated let-7f and miR-205 as biomarkers for the EOC diagnosis [275]. Since miRNAs with tissue-specific expressions are often stated for being dysregulated in cancer cells, they appear to be tumor indicators [276]. Several reports have revealed an unusual miRNA expression pattern as a prognostic indicator showing the disease result during the treatment. For example, in ovarian cancer cases, loss of let-7 expression along with the overexpression of HMGA2 is an indicator of a bad prognosis. Hence, the ratio of HMGA2 to let-7 is used to predict treatment outcomes; it means that cases with a higher ratio of HMGA2/let-7 have poor survival (<10%) in comparison with patients with a low ratio (40%) [241]. Furthermore, let-7 is underexpressed in chemotherapy-resistant patients and is associated with shorter survival; hence, it might be a potential biomarker for observing treatment outcomes and patients survival [240]. So, the miR-200 family was found to be a prognostic marker for ovarian cancer. Hu et al. studied 55 ovarian cancer patients with stages III and IV [277]. They found that expression of miR-200 family group, comprising miR-200a/b/c and miR-429, has been extremely reduced in patients with a recurrent cancer related to patients without recurrence. Eitan et al. in their research revealed the same conclusions [278]. They studied 57 patients with either serous or endometrioid histology who were at stage I or stage III of the disease and revealed downregulation of miR-200a, miR-34, and miR-449b miRNAs in advanced ovarian cancer (stage III) and high expression of miR-200a in initial stages of disease (stage I) with a more satisfactory conclusion. Also, studying 107 other patients from all types and stages disclosed higher survival in high-grade ovarian cancer patients mixed with high miR-200a levels in comparison to patients with lower expression of miR-200a [279]. miR-9 and miR-223 might be applicable as prognostic markers since they are correlated with the recurrent type of ovarian cancer [270]. Recently, the overexpression of miR-30d has been detected in patients that are sensitive to platinum-based chemotherapy compared to in chemoresistant patients. Moreover, the downregulation of miR-30d in patients with recurrent ovarian cancer has been noticed [280]. Lee et al. who were investigating miRNA expression in patients with ovarian cancer, benign ovarian cancers, and borderline malignancy discovered a higher expression of miR-30c, miR-30d, miR30e-3p, and miR-181d as survival prognostic markers [280]. Others have identified low expression of miR-34c and miR-422b in patients with a dropped survival of high-grade ovarian cancer [272]. Corney et al. indicated that downregulation of miR-34b/c, which is detected in the late stage of ovarian cancer, is related to mesenchymal-to-epithelial transition (MET) [271]. Kim et al. discovered a link between notable upregulation of miR-519a and poor survival [281]. miR-22 downregulated in ovarian cancer is correlated with overall survival and progression-free survival of patients; hence, it might be used as an efficient prognostic factor [282].

### 5.5. miRNAs as Novel Tools for Ovarian Cancer Diagnosis

miRNAs play a role as a perfect diagnostic and monitoring factor for ovarian cancer. Patients with a minimum level of miR-200 genes expression have poor survival with cancer growth [277]. Also, patients with low let 7a-3 methylation have minimal survival rate compared to patients with high methylation [202]. Precursor miR-146a causes G to C variants in patients with ovarian and breast cancer [283]. Ratio of HMGA2 to let-7 has been discovered as a prognosis factor in ovarian carcinoma patients [241]. The serum miRNAs are useful for recognition of cancer [273, 284]. Tumor-derived exosomes (small lipid vesicles) encompass diagnostic miRNAs [285]. Early detection of life-threatening malignancies, including ovarian cancer, has long been the key to successful therapy among the different considered preventive measurements [130]. The abnormal expression pattern of miRNAs expression can be useful for identifying an early-stage ovarian cancer. miR-200a and miR-141 were identified as exceedingly upregulated miRNAs in ovarian cancer, whereas miR-199a, miR-140, miR-145, and miR-125b1 were most astonishingly downregulated. In addition, to detect the histological types of ovarian tumors, dysregulation of a particular miRNA was also applied. For instance, there is an overexpression of miR-200a and miR-200c in all types (mucinous, endometrioid, and clear cells), miR-200b and miR-141 were overexpressed in serous as well as endometrioid carcinomas, and miR-21, miR-203, and miR-205 were overexpressed in endometrioid tumors. In fact, miR-145 is underexpressed in serous and clear-cell tumors, while miR-222 is underexpressed in endometrioid and clear-cell malignancies.

A different research has proven outstanding downregulation of serum miR-145 in malignant and benign ovarian cancers compared to healthy cases, and outcomes suggested that miR-145 can forcefully be used to discover ovarian and other types of human cancers [286]. Nam et al. determined 23 haphazardly expressed miRNAs in at least 60% of specimens of ovarian cancer with miR-21 as the most overexpressed miRNA (85% samples) and miR-125b (95% samples) as the most downregulated miRNA [236]. Yang et al. finalized 36 miRNAs differently expressed in normal and ovarian cancer cells, comprising miR-199a, miR-214, and miR-200a, respectively, overexpressed in 53, 56, and 43% of tumors of high-grade cancers [240]. miR-100 instead is downregulated in 76% of tumors. In comparison with previously mentioned data, Eitan et al. exhibited downregulation of miR-200a, miR-34a, and miR-449b in the evolved (stage III) ovarian tumors with miR-200a relation to the initial-stage cancer and enhanced overall survival [278]. Furthermore, miR-200a was discovered as a satisfactory outcome predictor in another research of 55 patients [277]. In addition, Marchini et al. recognized the correlation of miR-200c with progression-free survival or overall survival or both in phase I ovarian cancers [252]. The root cause for upregulation of miR-21, miR-203, and miR-205 in ovarian cancer tissues is hypomethylation of miRNA genes as the most essential epigenetic mechanism [132]. A different research has reported the overexpression of miRNAs as a consequence of miRNA genes amplification in tumors [233]. Among 35 miRNAs studied, there have been upregulation in four miRNAs (miR-26, miR-182, miR-103, and miR-26a) and downregulation in two miRNAs (let-7d and miR-127). These findings proposed alterations in copy number and epigenetic mediators as two reasons for unusual expression of miRNAs in ovarian cancer [233]. Thus, miR-30c, miR-30d, and miR-30e are frequently upregulated, whereas miR-493 is downregulated in ovarian tumors compared to in normal HOSE cell lines.

## 6. Implications of miRNAs in Targeting Ovarian Cancer

If ovarian cancer cells are confined to the ovaries, they can be effectively treated by debulking surgery. However, most of tumors are not diagnosed but are at more advanced metastatic stages of the disease, when chemotherapy treatment is required [287]. The majority of ovarian cancer cells respond effectively to neoadjuvant platinum-based chemotherapies. On the other hand, mesenchymal-like metastasis initiating cancer stem cells are usually resistant to these chemotherapies, resulting in cancer recurrence in many ovarian cancer patients [288]. Targeted therapy is another strategy in which crucial pathways for ovarian cancer cells growth or survival are blocked, while normal cells are not affected [289]. A possible targeted therapy for the treatment of ovarian cancer metastasis is based on targeting miRNAs. As described above, many miRNAs act as tumor promoters or suppressors in various cancers, and they can be given more attention as new therapeutic targets.

Thanks to the enhancement of cancer studies, patient-specific treatments soon would be developed. Since the expressions of a plethora of various genes are regulated by miRNAs, they can control and coordinate multiple cellular pathways [109, 130]. Therefore, miRNAs have been recommended as potential therapies against cancer. Correction of the mRNA expressions is an appealing strategy to target cancer cells, which can be done through the application of miRNA mimics (miRNA replacement therapy) to inhibit the upregulated onco-miRs by antisense miRs (miRNA inhibition therapy) [256]. miRNA drug MRX34, which is a miR-34 mimic, is the first and the only anticancer miRNA that has entered clinical trials in cancer patients. This miRNA mimic is being evaluated in clinical trials of phase I in hepatocellular carcinoma patients, which may finally open new avenues toward the application of miRNAs in cancer treatments.

Chen et al. suggested that the targeted delivery of miR-429 to carcinoma cells may have therapeutic effect for reducing cancer cell metastasis and tumor recurrence in advance-staged cancer patients [290]. miR-492 plays a significant role in controlling EMT and MET pathways; therefore, the overexpression of miR-429 may reduce metastasis by inducing epithelial phenotype in mesenchymal stem cell-like metastasizing cells reducing their tumorigenicity and metastatic potential [291]. Also, miR-492 is shown to be negatively correlated with resistance to Paclitaxel and cisplatin in ovarian adenocarcinoma cell lines [292]. Chen et al. have investigated the EOC cell lines, SKOV3 and COV644, and revealed the inhibition of metastasis and chemoresistance of EOC cells by miR-429 via KIAA0101-mediated Wnt/*β*-catenin signaling [184]. As a consequence, delivery of miR-429 inhibits the recurrence and improves survival rates of ovarian cancer.

The X-linked inhibitor of apoptosis protein (XIAP) acts as an apoptosis blockage and directly inhibits the effector caspases (caspases 3, 6, 7, and 9) [293] and promotes tumor formation and metastasis [294]. XIAP plays a crucial role in chemotherapy resistance in ovarian cancer and various tumors [295]. It is also highly expressed in ovarian cancer cells, which indicates the chemosensitivity of cancer cells [296]. XIAP is a direct target gene of miR-149 and miR-137. miR-137 [297] and miR-149 [298] are usually downregulated in ovarian cancer. The results obtained from several studies suggest that ectopic expression of miR-137 and miR-149 can suppress the XIAP 3′UTR function and decrease the levels of XIAP protein in ovarian cancer cells [297–299]. miR-150 is considered as an EMT process regulator but is downregulated in primary EOC tissues [234], and ectopic expression of miR-150 blocks proliferation, invasion, and metastasis of EOC cells [300].

miR-320, which is a tumor suppressor, is markedly downregulated in EOC cells and cell lines. *MAPK1* [301] and *TWIST* [302] are two direct target genes of miR-320 in EOC, which are significantly upregulated in EOC tissues, and their downregulation represses the proliferation and invasive ability of EOC cells. MAPK1 plays a crucial role in the MAPK/ERK pathway and mediates various biological processes, including cell growth, cell survival, and differentiation through the regulation of transcription, translation, and cytoskeletal rearrangements [303, 304]. Therefore, decreased expression of MAPK1 suppresses SKOV3 cells growth and invasion in ovarian cancer [305]. TWIST1 is one member of the basic helix-loop-helix transcription factor twist family that plays important roles in the mesenchymal phenotype, serves as one potent oncogene [306], and is overexpressed in various cancers. Overexpression of TWIST1 promoted tumor cell migration, proliferation, and invasion [307]. Besides, miR-186 is another miRNA that inhibits EMT in ovarian cancer cells and stimulates G1 cell-cycle arrest and promotes apoptosis by regulating TWIST1 [308].

miR-34 family is thought to be underexpressed in EOC, especially those with mutations in *TP53*, and was linked to tumor stage [271]. It is also demonstrated that replacement therapy with miR-34 in SKOV3 cell line has drastically reduced proliferation, migration, and invasion. In most of ovarian cancers, miR-200 family members (miR-141, miR-200a, miR-200b, miR-200c, and miR-429) are underexpressed correlating with poor survival [132, 236]. The miR-200 family inhibit EMT, cell migration, and metastasis through impacting on ZEB1 (zinc-finger E-box-binding homeobox 1) and ZEB2 [279, 309]. Chen et al. reported that IKK expression is regulated by miR-199a, which changes the inflammatory microenvironment in ovarian cancer [310]. Epigenetic silencing of miR-199b-5p is linked to chemoresistance in ovarian cancer, since it activates JAG1/Notch1 signaling [311]. Recently, meta-analysis of the transcriptome of high-grade serous ovarian carcinoma (HGSOC) has revealed that let-7b, as a weak prognostic marker, helps predict molecular and clinical classes of HG-SOC [312].

Studies have reported that miR-200 affects interleukin-8 (IL-8) and CXCL1 in epithelial cancer cells, thereby inhibiting angiogenesis, demonstrating the therapeutic role of miR-200 in ovarian cancer treatment [313]. It is also found that loss of miR-31 results in TP53 pathway deficiency, highlighting the potential therapeutic benefits of this miRNA in patients with *TP53* deficient activity [124, 262]. Taken together, since a variety of human cancer cells, including ovarian cancer cells, have been shown to have altered expression of different miRNAs, the development of a targeted therapeutic agent that would suppress or provoke oncogenic/tumor suppressor miRNAs would be a promising antitumor therapy against cancer.

## 7. Conclusions

Regardless of the advancements achieved for prognosis, diagnosis, and monitoring of ovarian cancer, most of the patients succumb to this devastating malignancy, mostly because of the late-stage diagnosis and recurrent disease. Therefore, the need for identifying reliable markers for early detection and EOC patients monitoring, which are both sensitive and specific, remains a long-awaited priority. EOC management could be well supported by the application of the biomarkers for discriminating malignant tumors from benign pelvic masses, early diagnosis, estimating prognosis, monitoring the treatment efficacy, and predicting response to individual drugs. Current screening and monitoring indicators for ovarian cancer do not meet the requirements for fulfilling cancer diagnosis, tumor subtype classification, chemoresistance monitoring, and outcome prognosis. For instance, carcinoembryonic antigen (CEA) is a broad-spectrum biomarker for diagnosis of various types of cancers and lacks sensitivity. CA125, which is the most well-known biomarker for EOC screening, lacks sensitivity, especially for the diagnosis of early-stage patients and could not discriminate the malignant tumors from benign pelvic cysts. Human epididymis protein 4 (HE4), which is another marker usually applied for EOC detection, lacks acceptable specificity and could not monitor the tumor burden during the treatment course. Therefore, incorporating other types of biomarkers, either alone or in combination with the traditional biomarkers, may help ameliorate the diagnosis and monitoring of the patients. Considering the miRNAs as biomarkers for prognostication and screening of cancer patients is promising, because they usually exhibit aberrant expression under different pathological conditions, including ovarian malignancy. miRNAs are involved in various cellular processes. Therefore, a plethora of these markers are available, making it possible to trace any aspect of cellular and molecular mechanisms involved in cancer progression. In addition, miRNAs benefit from structural stability, making them promising for being considered as screening and monitoring biomarkers (Table 2). Furthermore, since each miRNA usually targets a vast number of mRNAs, targeting or the delivery of miRNAs may be an interesting approach for efficiently targeting ovarian cancer cells. Further studies are, however, needed to suffice the role of miRNAs as biomarkers in clinical settings for ovarian cancer management.

## Figures and Tables

**Table 1:** Targets and functions of important miRNAs associated with ovarian cancer.

miRNA	Target	Alteration	Cellular function	Ref.
miR-214	Sema 4D (Semaphoring 4D)	Upregulation	Inhibits cell proliferation and promotes apoptosis	[137]
	LHX6 (LIM Homeobox 6)	Downregulation	Induces apoptosis	[138]
	PETN (phosphatase and tensin homolog)	Upregulation	Promotes radioresistance	[139]
miR-182	BNIP3 (BCL2 interacting protein 3)	Upregulation	Inhibits proliferation and migration	[140]
miR-23b	CCNG1 (cyclin G1)	Upregulation	Inhibits ovarian cancer tumorigenesis and progression	[141]
miR-381	YY1 (the ubiquitous transcription factor yin yang 1)	Upregulation	Inhibits ovarian cancer cell proliferation, migration, and invasion	[142]
miR-132	E2F5 (transcription factor)	Upregulation	Inhibits cell migration and invasion of cancer cells	[143]
miR-143-3p	TAK1 (transforming growth factor-*β*-activated kinase 1)	Upregulation	Inhibits the proliferation, migration, and invasion of ovarian cancer cells	[144]
	CTGF (connective tissue growth factor)	Upregulation	Inhibits ovarian cancer cells proliferation, migration, and invasion	[145]
miR-145	CCND2 & E2F3 (cyclin D2)	Upregulation	The invasion and proliferation suppressor	[146]
miR-34a-5p	NEAT1 (nuclear paraspeckle assembly transcript 1)	Downregulation	Suppresses Cell proliferation and triggers apoptosis	[147]
miR-383-5p	TRIM27 (tripartite motif containing 27)	Upregulation	Proliferation, chemosensitivity suppressor and enhancer in ovarian cancer cells	[148]
miR-148a-3p	c-Met (tyrosine-protein kinase *Met*)	Upregulation	Inhibits invasion of ovarian cancer cells and cell migration	[149]
miR-141	KEAP1 (Kelch-like ECH-associated protein 1)	Upregulated	Cisplatin resistance	[150]
miR-218	RUNX2 (runt-related transcription factor 2)	Upregulated	Inhibits proliferation and invasion	[151]
miR-199a	HIF-1*α* & HIF-2*α* (hypoxia-inducible factor)	Upregulation	Decreases migration and metastatic tumor formation	[152]
miR-491-5p	EGFR (epidermal growth factor receptor) and *BCL*-X_L_	Upregulation	Induces cancer cell apoptosis by blocking downstream AKT and MAPK signaling pathways	[153]
miR-744-5p	HNRNPC (heterogeneous nuclear ribonucleoproteins C1/C2) and NFIX (nuclear factor 1 X-type)	Upregulation	Induces tumor cell apoptosis	[154]

**Table 2:** Significant miRNAs with potential prognostic and diagnostic biomarkers.

Biomarker	Prognosis	Diagnosis	Tumor stages	Endpoint	Source	Ref.
miR-532-5p	Yes	No	Cell proliferation of ovarian neoplasms	OS	—	[242]
miR-200b	Yes	No	EMT and migration	OS	Ascitic fluid	[243, 244]
miR-193b	No	Yes	Lymph node metastasis	Poor survival	Tissue	[245]
miR-135a-3p	Yes	No	Cell proliferation of ovarian tumors	Decreased PFS	Peritoneal fluid	[246]
miR-125b	Yes	Yes	Lymph node involvement and distant metastasis	Decreased OS	Serum	[247]
miR-613	Yes	No	Lymph node involvement	Short PFS and OS	Tissue	[248]
miR-506	Yes	No	EMT	Decreased OS and PFS	Tissue	[249]
miR-205	Yes	Yes	Advanced stages III/IV	—	Plasma	[250]
miR-200c-3p, miR-346, miR-127-3p	No	Yes	Distant metastasis	—	Serum	[251]
miR-200c	No	Yes	Stage I	OS and PFS	Tissue	[252]
miR-27a and miR-23a	Yes	No	Stages I-IV	OS, RFS, and short PFS (miR-27a)	Serum	[253]
miR-let-7	Yes	No	Early tumor progression	Poor survival	Tissue	[241]
miR-221	Yes	Yes	Stages I–IV	Decreased OS	Serum	[254]
miR-21	Yes	Yes	Stages I–IV	OS	Serum	[255]
miR-335	Yes	No	Distant metastasis (stages III/IV)	OS and RFS	Tissue	[256]
miR-429	Yes	Yes	Stages III-IV	OS	Serum	[168]
miR-199a	Yes	Yes	Stages III-IV	Poor OS		[257]
miR-1246	No	Yes	Stages III-IV	—	Serum	[258]

PFS: progression-free survival, OS: overall survival, RFS: recurrence-free survival.

## Data Availability

There are no raw data associated with this review article.
